# Vasculogenic mimicry in carcinogenesis and clinical applications

**DOI:** 10.1186/s13045-020-00858-6

**Published:** 2020-03-14

**Authors:** Qingxi Luo, Jun Wang, Wenyuan Zhao, Zhenzi Peng, Xianyu Liu, Bin Li, Heng Zhang, Bin Shan, Chunfang Zhang, Chaojun Duan

**Affiliations:** 1 Department of Thoracic Surgery, Xiangya Hospital, Central South University, Xiangya Road 87th, Changsha, 410008 Hunan People’s Republic of China; 2 Department of Oncology, National Clinical Research Center for Geriatric Disorders, Xiangya Hospital, Central South University, Changsha, 410008 People’s Republic of China; 3 College of Medicine, Washington State University Spokane, Spokane, WA 99201 USA

**Keywords:** Vasculogenic mimicry, Cancer, Mechanisms, Molecular imaging technologies, Tumor therapy

## Abstract

Distinct from classical tumor angiogenesis, vasculogenic mimicry (VM) provides a blood supply for tumor cells independent of endothelial cells. VM has two distinct types, namely tubular type and patterned matrix type. VM is associated with high tumor grade, tumor progression, invasion, metastasis, and poor prognosis in patients with malignant tumors. Herein, we discuss the recent studies on the role of VM in tumor progression and the diverse mechanisms and signaling pathways that regulate VM in tumors. Furthermore, we also summarize the latest findings of non-coding RNAs, such as lncRNAs and miRNAs in VM formation. In addition, we review application of molecular imaging technologies in detection of VM in malignant tumors. Increasing evidence suggests that VM is significantly associated with poor overall survival in patients with malignant tumors and could be a potential therapeutic target.

## Background

It is widely acknowledged that solid tumors need sufficient blood supply for growth. When the diameter of a solid tumor is greater than 2 mm, the formation of new blood vessels is necessary to maintain sufficient blood supply; otherwise, tumors will undergo necrosis due to ischemia and hypoxia [1]. Tumor-induced angiogenesis involves the release of various angiogenic factors such as vascular endothelial growth factor (VEGF), which causes morphological changes in vascular endothelial cells, the basement membrane, and surrounding extracellular matrix. In 1999, Maniotis et al. first proposed the concept of vasculogenic mimicry (VM) in human melanoma. VM is a new tumor microcirculation model and distinct from classical tumor angiogenesis because it does not depend on endothelial cells and can provide sufficient blood supply for tumor growth [2]. Moreover, VM is associated with high tumor grade, invasion, metastasis, and poor prognosis in patients with malignant tumors [3–6]. In recent years, VM has been reported in a variety of malignant tumors, such as melanoma, glioblastoma, osteosarcoma, hepatocellular carcinoma (HCC), breast cancer, lung cancer, gastric cancer, colorectal cancer, and prostate cancer [7–19]. VM has emerged as a promising new target for anti-tumor therapy [20, 21]. There are several potential mechanisms of VM formation, such as epithelial-mesenchymal transition (EMT) and cancer stem cells (CSCs) [22, 23], and various signaling pathways that promote VM formation, including vascular endothelial (VE)-cadherin, erythropoietin-producing hepatocellular receptor A2 (EphA2), phosphatidyl inositol 3-kinase (PI3K), matrix metalloproteinases (MMPs), vascular endothelial growth factor receptor (VEGFR1), cyclic adenosine monophosphate (cAMP), focal adhesion kinase (FAK), and hypoxia inducible factor (HIF)-1a [24, 25]. Moreover, non-coding RNAs such as lncRNAs and miRNAs play critical roles in VM formation in malignant tumors [16, 26–28]. In this review, we provide new insights into the complexity of vasculogenic mimicry and summarize the latest findings of VM formation in malignant tumors.

## Different forms of tumor angiogenesis

**Fig. 1:**
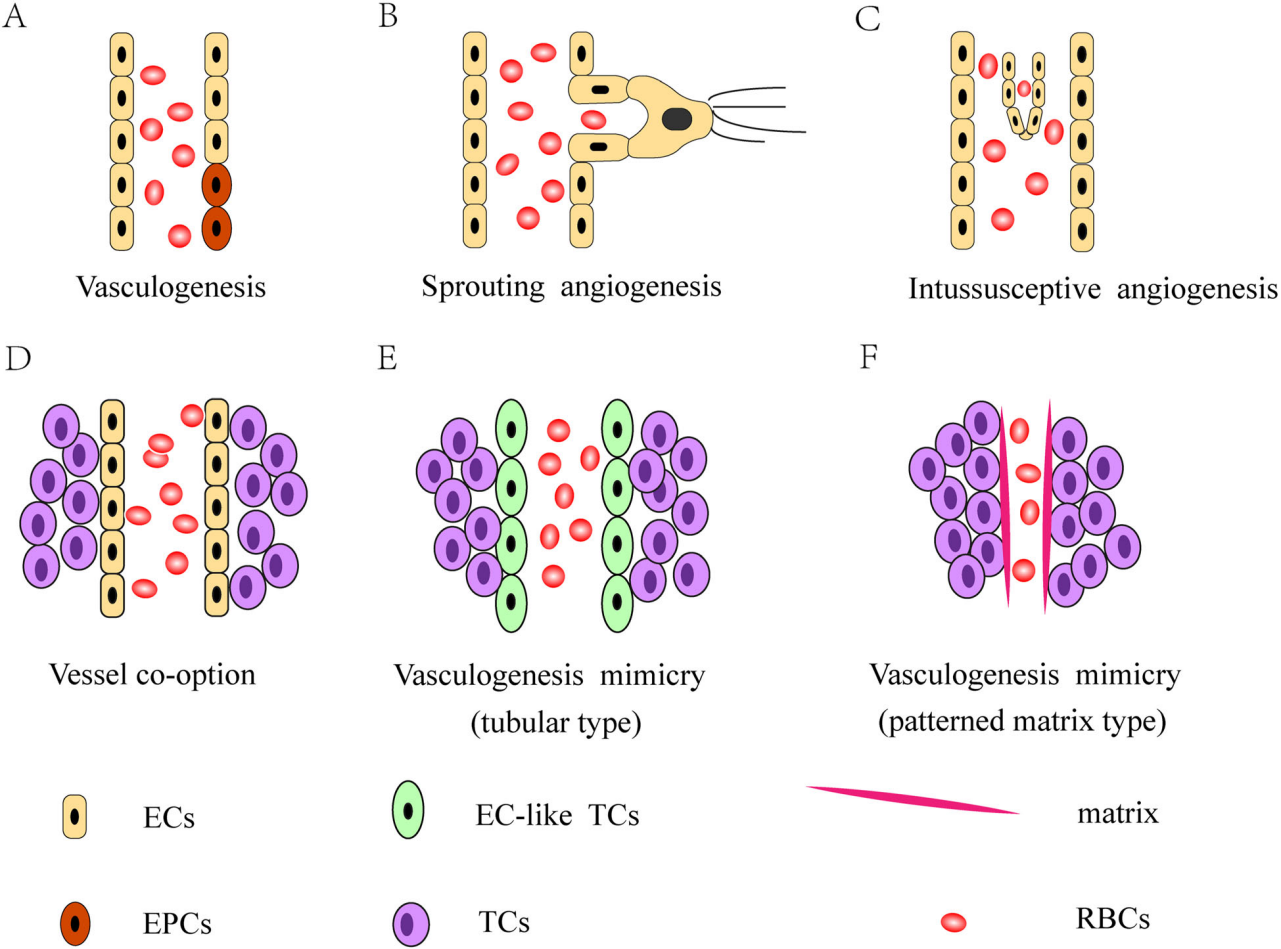
Different forms of tumor angiogenesis. **a**Vasculogenesis: recruiting endothelial progenitor cell (EPC) to participate in the formation of tumor blood vessels. **b** Sprouting angiogenesis: the sprouting of new blood vessels from the existing vasculature. **c** Intussusceptive angiogenesis: the splitting of pre-existing vessel to give rise to daughter vessels. **d** Vessel co-option: hijacking the existing vasculature and tumor cells migrate along the existing or newly induced blood vessels. **e**, **f** Vasculogenic mimicry: does not depend on endothelial cells, VM of tubular type are lined by EC-like tumor cells and covered by secretory glycoprotein, and the patterned matrix type is covered by the PAS-positive matrix.

The formation of tumor blood vessels can occur in a variety of ways, including vasculogenesis, angiogenesis, intussusceptive angiogenesis, vessel co-option, and vasculogenic mimicry. Vasculogenesis and angiogenesis are the main mechanisms of tumor angiogenesis. Vasculogenesis is achieved through the recruitment of endothelial progenitor cells (EPCs) that are capable of differentiating into endothelial cells and migrating to the tumor to directly participate in the formation of tumor blood vessels. Angiogenesis refers to the origination of tumor blood vessels from existing endothelial cells and the formation of new neoplastic capillaries by sprouting. Angiogenesis is the most widely investigated mode of new vessel formation in tumors [29]. Intussusceptive angiogenesis (IA) occurs in the lumen of existing blood vessels and is mediated by the interstitial columnar structure, resulting in the segmentation of the original vascular lumen and the formation of new blood vessels, which splits pre-existing vessels to give rise to new vessels [30, 31]. Vessel co-option may occur in many malignancies, which means hijacking the existing vasculature and tumor cells migrate along the existing or newly induced blood vessels to supply tumor growth and metastasis [32, 33]. Vasculogenic mimicry refers to a new tumor microcirculation model that is distinct from the classical tumor angiogenesis pathway and does not depend on endothelial cells [2] (Fig. 1).

Under pathological conditions, tumor angiogenesis is an extremely complex process. First, tumor cells release angiogenic factors that cause a disturbance in the balance between pro- and anti-angiogenic factors in the tumor vascular microenvironment. Second, the basement membrane underneath endothelial cells is degraded by diverse molecules. Last, endothelial cells are recruited to the corresponding sites by chemokines and pro-angiogenic factors, and the new blood vessels are formed by sprouting branches from the original blood vessels [31, 34, 35]. The reason why tumors are prone to invasion and metastasis is that tumor vascular structure and function are abnormal, and the vascular matrix is imperfect, which results in easy penetration by tumor cells. Tumor cells can penetrate the blood vessels and form a metastatic lesion at a distant site. Many molecules and cell types participate in the process of tumor angiogenesis, such as vascular endothelial growth factor (VEGF), fibroblast growth factor-2 (FGF-2), matrix metalloproteinases (MMPs), tumor-associated macrophages (TAMs), and tumor-associated fibroblasts (TAFs) [30, 36]. Recently, emerging evidence indicates that non-coding RNAs play crucial roles in tumor angiogenesis [29, 37].

VM has been categorized in two distinct types: tubular type and patterned matrix type. VM of the tubular type features tubular channels that are lined by tumor cells rather than endothelial cells and are covered by secretory glycoprotein. A number of mechanisms of tubular type formation have been reported, such as the sculpting of pathways through tumors and the invasion of the walls of vessels by tumor cells [38–40]. In glioblastomas, when there is insufficient blood supply in the tumor, glioblastoma stem-like cells express pro-vascular molecules such as EGFR, endothelium-associated genes including EphA2, Laminin 5γ2, and Neuropilin-2, leading to the de novo formation of blood vessels with GSCs as the lining cells of the luminal surface [41]. The patterned matrix type features tumor cells and tissue that are wrapped around by PAS-positive matrix proteins such as laminin, heparan sulfate proteoglycan, and collagens IV and VI [42]. This type has neither the tubular nor ultrastructural characteristics of blood vessels, which could be precisely described by its extravascular matrix patterns [40]. It has been reported that highly invasive melanoma cells damage vessels to allow the flow of plasma and red blood cells to enter the adjacent patterned matrix [40, 43]. The formation of VM mainly includes self-deformation of highly malignant tumor cells, remodeling of the extracellular matrix, and a resulting vascular-like structure that is connected with the existing blood vessel. Thus tumor cells and tissues obtain blood supply [2, 40]. The criteria of the VM structure are as follows: (1) absence of vascular endothelial cells on the inner wall of the VM blood vessel; (2) vascular-like channels are lined with tumor cells; (3) positive for PAS staining but negative for CD31 staining, while the endothelial vascular channels are negative for PAS staining but positive for CD31 staining; (4) erythrocytes in the vascular-like channels [44, 45]. Patient-derived xenografts models have been used to dissect the mechanisms responsible for VM and tumor progression. In melanoma, NOD/SCID and RAG2−/− mice were injected melanoma cells isolated from melanoma patients, and the results showed PAS-positive and CD31-negative channel formation in humanized xenografts [46]. Prevalent circulating tumor cells (CTCs) is one of the characteristic of small cell lung cancer (SCLC). In 2016, Stuart C et al. reported that VM is present in CTC patient-derived explants (CDX) models and VM is confirmed to be of human tumor origin [19].

## Mechanisms of tumor VM formation

VM formation is an extremely complex process that involves a variety of complex mechanisms.

### Epithelial-mesenchymal transition and VM

EMT is the process by which epithelial cells are transformed into mesenchymal phenotype cells via specific steps, including reduction of expression of cell adhesion molecules (such as E-cadherin), transformation of cytokeratin cytoskeleton into vimentin-based cytoskeleton, and acquisition of morphological characteristics of mesenchymal cells [29]. EMT plays a very important role in VM-forming tumor cells and promotes tumor invasion and metastasis through various mechanisms [43]. In this process, some epithelial cell marker proteins are downregulated, such as E-cadherin, zonula occludins-1, and α-catenin. On the other hand, some of mesenchymal cell markers are upregulated, including VE-cadherin, fibronectin, cadherin-2, and vimentin. VE-cadherin is a VM biomarker and plays a crucial role in the process of VM formation [23, 43]. Some EMT-associated transcription factors including Snail1, Slug (Snail2) [45], Zinc finger E-box binding homeobox 1 (ZEB1) [18, 46], ZEB2 [13] and Twist1 [11]. These transcription factors can bind to the E-cadherin promoter to regulate transcriptional activity, and the decreased levels of E-cadherin result in decreased cell adhesion and increased cell invasion and metastasis [22, 30]. EMT involves a large number of signaling pathways, such as the transforming growth factor-beta (TGF-β) signaling pathway [13, 47], Wnt signaling pathway [48, 49] and Notch signaling pathway [31, 32, 50, 51]. Furthermore, there are two miRNA regulatory networks that are considered prominent regulators of EMT: the miR34-SNAIL1 and miR200-ZEB1 axes that regulate EMT epigenetically [47]. Research has shown that snai1 and slug regulate the process of EMT by repressing E-cadherin transcription to disrupt cell-to-cell adhesion. Furthermore, reactive oxygen species; (ROS) can activate Snai1 to promote cancer progression [48]. It has been demonstrated that slug expression was significantly associated with the CSCs phenotype and VM formation in HCC [49]. ZEB1 inhibits the transcriptional of E-cadherin by recruiting multiple chromatin enzymes to the E-cadherin promoter, and cause the cells to lose epithelial properties [50]. ZEB2, a homologous protein of ZEB1, also induces EMT. Yang et al. found that ZEB2 expression could increase tumor cell invasion and migration upregulate VE-cadherin expression and activate MMPs to promote VM formation in hepatocellular carcinoma [13]. Twist1 induces EMT by affecting the expression from the E-box containing promoters at the transcriptional level, thereby inhibiting the expression of E-cadherin. Low expression of E-cadherin leads to a decrease in membrane-bound β-catenin, an increase in intracellular free β-catenin, and an increase in vimentin expression. In addition, Sun et al. reported that twist1 expression was associated with VM formation via regulating VE-cadherin transcription and MMP activation [51–53]. Furthermore, a common feature of these EMT inducing transcription factors is that their DNA-binding motifs recognize the E-box motif on the promoter of the target genes. Transforming growth factor-β (TGF-β) is a highly expressed cytokine in the tumor microenvironment and induces the expression of a large number of genesin tumor cells as the best known regulator of EMT. Yang et al. showed that E-cadherin protein decreased and VE-cadherin increased in TGF-β1-treated cells and ZEB2 showed higher expression after TGF-β treatment, whereas TGF-β had no significant effect on the protein expression of transcription factors, including twist1, snail1, and slug [13]. Wnt signaling is involved in several physiological processes, such as cell proliferation, endothelial cell differentiation, abnormal vascular development, and angiogenesis. Furthermore, Wnt signaling induces EMT by inhibiting glycogen synthase kinase-3β (GSK3β)-mediated phosphorylation and inhibition of β-catenin degradation in cytoplasm. Wnt ligands, such as Wnt1, Wnt3a, and Wnt7a, are glycosylated in the ER (endoplasmic reticulum) and Golgi and transported through the Golgi to the plasma membrane. Cell-bound-Wnts may spread over tissues via cell division. Previous reports have shown that Wnt signaling plays an important role in vascular development and angiogenesis [12, 54, 55]. In colon cancer, Qi et al. reported that increased Wnt3a expression and β-catenin nuclear expression are associated with VM formation [55]. In addition, it was reported that Wnt5a might enhance VM formation via the PKCalpha pathway in epithelial ovarian cancer [56].

### Tumor microenvironment and VM

#### Cancer stem cells

CSCs are defined as cells that have self-renewal ability and produce heterogeneous tumor cells in tumors. CSCs were first demonstrated in human acute myeloid leukemia (AML) in 1994. Lapidot et al. found that AML cells grafted into SCID mice produce a large number of colony-forming progenitor cells that are a CD34+/CD38− subpopulation of leukemic cells [57]. CSCs are also found in a number of solid tumors, including breast cancer, prostate cancer, brain cancer, lung cancer, and colon cancer [58–60]. CSCs may be the source of all the tumor cells present in malignant tumors, as well as the cause of distant metastasis and VM formation of tumors. CSCs have the ability to differentiate/transform VM and align to form a branching stream that provides nutrients to the tumor mass [23, 61]. It has been reported that a number of CSC markers promote invasion and metastasis in breast cancer stem cells [62]. Emerging evidence indicates that CSCs are involved in VM formation. Human triple-negative breast cancer (TNBC) corresponds to the basal-like subtype of breast cancer that is negative for estrogen receptor (ER), progesterone receptor (PR), and human epidermal growth factor receptor 2 (HER2). Liu et al. found that a CSC subpopulation with holoclone morphology, CD133^+^ status (a putative CSC marker), phenotype, and CSC characteristics was positively associated with VM in TNBC. In the same study, they explored the relationship between CD133 expression and VM. Their findings indicate that VM is critical for TNBC relapse and progression [63, 64]. Furthermore, Yuki Izawa et al. demonstrated that ALDH+ cells (a subpopulation of CSCs) exhibit more VM formation on Matrigel than ALDH− cells. These results confirmed CSCs as a crucial modulator in the process of VM formation [65]. In glioblastoma, glioblastoma stem like cells (GSCs) that express endothelial cell marker CDH5. CDH5 was specifically upregulated in GSCs but not non-GSCs or neural stem cells. These cells may contribute to the VM formation, especially under hypoxic condition [9]. Wu et al. revealed that bevacizumab-induced autophagy plays a crucial role in the formation of VM in GSCs [66]. ALM201, a FKBPL-derived therapeutic peptide, could reduce CSCs through inducing differentiation and target CSCs in high-grade serous ovarian cancer (HGSOC) cell lines, but ALM201 was unable to do so in non-vascularised OVCAR3 xenografts due to VM [67]. Additionally, in prostate cancer, ZEB1 regulates VM formation and alters the expression of CSC-associated protein CD133. Furthermore, the follow-up experiments revealed that ZEB1 knockdown reduces the level of p-Src [527], the results showed ZEB1 is essential to CSC phenotype and VM formation by Src signaling [18]. Recently, in hepatocellular carcinoma, Zhao et al. found that LncRNA n339260 promotes VM formation in HCC by inducing CSC-like phenotype and the knockdown of n339260 reduced VM and CSCs. This study suggested that the molecular target of n339260 may offer a promising new therapy [68].

#### Tumor-associated macrophages

In the past, it was thought that macrophages could directly kill tumor cells or induce the immune response during the anti-tumor immunomodulation process. Recently, some studies have shown that tumor-associated macrophages do not play an anti-tumor role, but promote tumorigenesis, growth, invasion, migration, and VM formation [69–71]. In 2016, Barnett et al. reported that macrophages play a supportive role in the process of the formation of a primitive tubular network in tumors and Matrigel. They found that the tubular structure is not an endothelial cell-lined tubular channel as indicated by transmission electron microscopy (TEM) and scanning electron microscopy (SEM). Further experiments revealed HIF-1α is an important driver in this process of VM network formation [69]. Rong et al. showed that M2 macrophages could promote VM formation via the PGE2/EP1/PKC pathway in glioblastoma multiforme (GBM) in a COX-2 dependent manner [72]. In 2017, Zhang et al. reported M2-like macrophages promoted VM formation by amplifying IL-6 secretion via the PKC pathway in vitro studies of glioma cell lines [70].

#### Tumor-associated fibroblasts

**Fig. 2:**
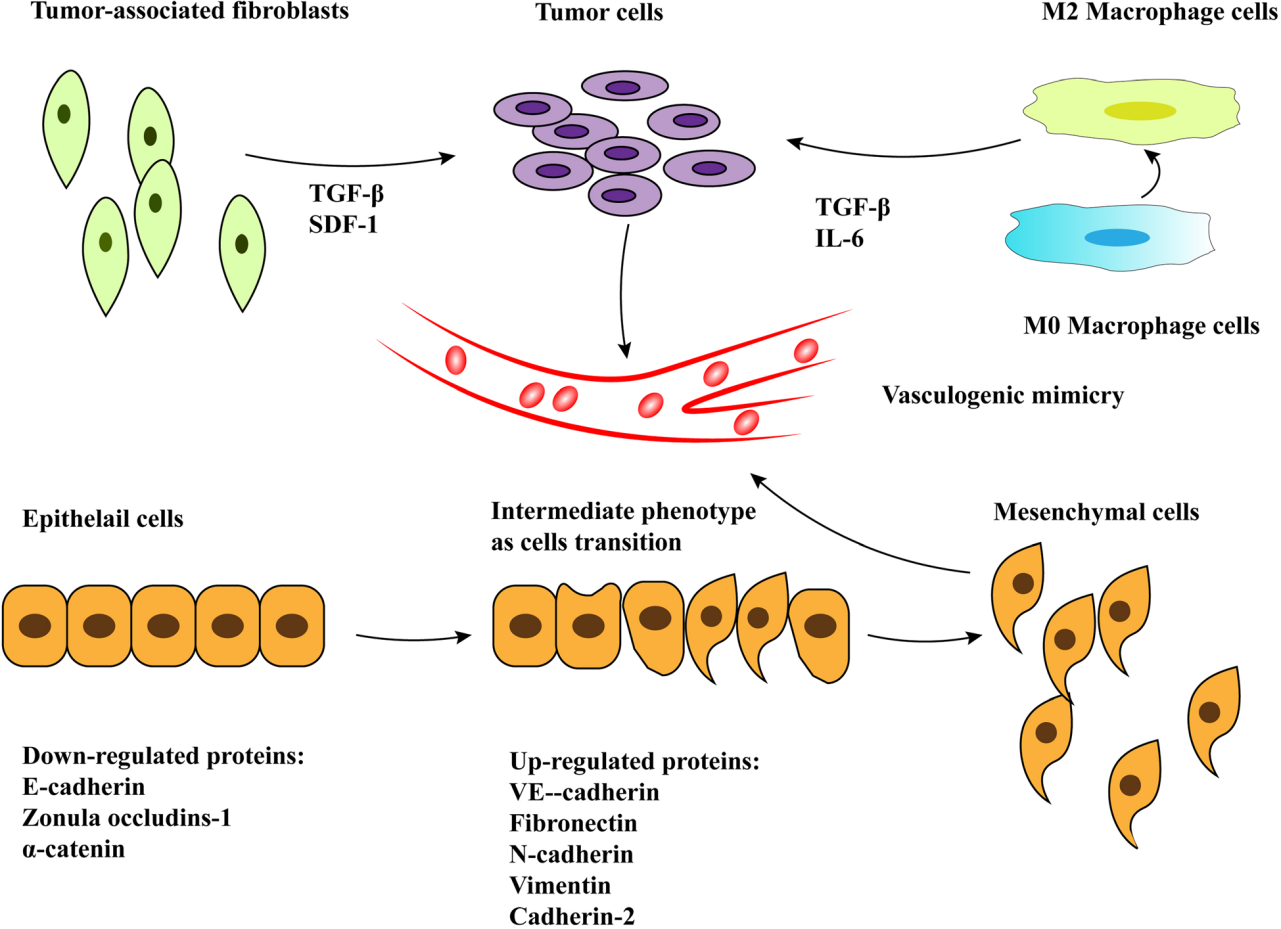
(1) Tumor microenvironment and VM: TAFs (tumor-associated fibroblasts) promote VM formation through secreting TGF-β and SDF-1, and TAMs (tumor-associated macrophages) promote VM formation by amplifying IL-6and TGF-β secretion. (2) The process of EMT participating in the VM formation: epithelial cells are transformed into mesenchymal phenotype cells by specific procedures, including reduction of expression of cell adhesion molecules (such as E-cadherin) and some upregulated proteins (such as VE-cadherin-biomarker of VM).

Tumor-associated fibroblasts are the activated fibroblasts isolated from the tissues of tumor patients and characterized by the expression of fibroblast-activated proteins (FAP) and α-SAM. TAFs are important factors in tumor growth and VM formation [73–75]. Some studies have demonstrated that TAFs are required for tumor vascularization via various signaling pathways in malignant tumors [76–78]. In 2016, Yang et al. showed that the conditioned medium of tumor-associated fibroblasts (CM-TAMs) could promote VM formation in vitro through secreting TGF-β and SDF1, and the expression of VE-cadherin, MMP-2, and laminin5γ2 was increased by TGF-β and SDF1 in hepatocellular carcinoma [73]. Kim et al. investigated the relationship between EphA2 and VM formation induced by cancer-associated fibroblasts (CAFs) in gastric cancer. They revealed that CAF-CM-induced VM formation was blocked by EphA2-inhibitor and PI3K-inhibitor. In conclusion, CAFs regulate VM formation via EphA2-PI3K signaling in gastric cancer cells [79] (Fig. 2).

#### Hypoxia

The hypoxia microenvironment is a common situation in growing tumors. In tumor tissues, the uncontrolled growth and proliferation of tumor cells consume a large amount of nutrients and oxygen. It is well known that the vast majority of solid tumors exist in the hypoxic microenvironment. Hypoxia is one of the most important factors in VM formation [68]. Hypoxia-inducible factors (HIFs) and hypoxia responsive elements (HREs) play an important role in this context. Some hypoxia responsive genes containing HREs are involved in VM, such as Twist, E-cadherin, Nodal, EphA2, and VEGF-A [5]. In human melanoma, there is a positive correlation between the VM formation and the ischemic group and the expression of HIF-1a, and HIF-1a expression is positively correlated with VEGF expression [68]. In human ovarian carcinoma, HIF-1a promotes VM formation by inducing a shift in expression from E-cadherin to vimentin [72].

### Key molecules and signaling pathways in tumor VM formation

VM associated molecules and signaling pathways have been investigated in numerous types of highly aggressive malignant tumors in recent studies.

#### EphA2

Erythropoietin-producing hepatocellular receptor (EphA2) is a transmembrane tyrosine kinase receptor and a member of the tyrosine kinase receptor (RTK) superfamily. EphA2 was found to be expressed only in highly aggressive malignant tumors, and associated with VM formation [1–3]. A recent report showed that miR-141 may regulate VM formation by controlling EphA2 expression in human glioma. The EphA2 3′UTR contains seven potential miR-141 binding sites. A luciferase reporter assay indicated that EphA2 serves as a miR-141′s target in glioma cells, and that there is a negative correlation between miR-141 and the expression of EphA2. In conclusion, EphA12 is a potential regulator in the development of VM [1]. In head and neck squamous cell carcinoma (HNSCC), knocking down EphA2 in vitro leads to a reduction in the number of VM channels, and the expression of EphA2 and EMT-related markers such as Twist and Vimentin expression have a positive association [2]. In gallbladder carcinomas, upregulation of PI3K/MMPs/Ln-5γ2 and/or EphA2/FAK/paxillin contributed to tumor growth and VM formation [3]. In 2019, Yeo et al. showed that serum induces tumor invasion and VM formation through the EphA2/VE-cadherin/AKT pathway, and upregulates the expression levels of VE-cadherin, matrix metalloproteinase-2 (MMP-2), and laminin subunit 5 γ-2 (LAMC2) protein [4].

#### FAK

Focal adhesion kinase (FAK), a cytoplasmic tyrosine kinase, plays an important role in tumor progression. It has been reported that the FAK signaling pathway plays an important role in regulating VM formation in aggressive malignant tumor cells. Zang et al. found that in gastric cancer overexpressing CEACAM6 (carcinoembryonic antigen-related cell adhesion molecule 6) can increase the phosphorylation of FAK and its downstream target paxillin, and then promote angiogenesis and VM formation [5]. In 2017, Zhou et al. reported that CDK5 (Cyclin-dependent kinase 5) kinase induces VM formation in non-small-cell lung cancer by activating of the FAK and AKT signaling pathways. They found that in lung cancer cells FAK knockdown damages cytoskeleton and microfilament formation which are essential toVM formation [6]. It has been reported that phosphoVEC (pVEC) as a target of FAK and a component of the Kaiso transcriptional complex that mediates VM in human malignant melanoma cells [7].

#### PI3K

Phosphatidylinositol 3-kinase (PI3K) is an intracellular phosphatidylinositol kinase that is involved in the production of oncogenes such as V.SRC and V.RAS. PI3K plays an important role in the uncontrolled cancer cell growth [8]. The importance of the PI3K pathway in the process of VM formation has been reported. Recently, experimental evidence has shown that the PI3K/MMPS/Ln-5γ2 signaling pathway mediates VM formation in aggressive human gallbladder carcinomas. In this study, the expression of VM signal-related proteins was effectively inhibited by TIMP-2, and the formation of vasculogenic-like structure consequently decreased. It is known that the proteolytic activities of MMP-2 and MT1-MMP (membrane type 1 matrix metalloproteinase) can be effectively inhibited by TIMP-2. Inhibition of PI3K may block the cleavage of the Ln-5γ2 chain and reduce MMP-2 and MT1-MMP [3]. LR1G1 as a negative regulator of the EGFR/PI3K/AKT signaling pathway inhibits hypoxia-induced VM formation. LR1G1 overexpression reduces the expression of phosphorylated EGFR (pEGFR), PI3K (pPI3K), and AKT (pAKT), and EMT biomarkers such as E-cadherin and vimentin [9].

#### MMPs

Matrix metalloproteinases (MMPs) are a large family with 26 members. MMPs play a key role in tumor invasion, metastasis, and VM formation. They are considered to be the main proteolytic enzymes in this process. The expression of high levels of MMPs is one of the most important prerequisites for VM formation. Highly aggressive tumor cells express high levels of MMPs (MMP-1, MMP-2, and MMP-9), and the 5γ2 chain of laminin. Activated MMPs and laminin could promote the formation of blood vessels via VM [10]. In melanoma tumor cells, PI3K plays an important role in the process of VM formation by affecting MMP-2 and MT1-MMP activity. Several studies documented that the cleavage process of laminin 5γ2 chain was blocked after inhibition of PI3K, resulting in decreased levels of the laminin 5γ2′ and laminin 5γ2x fragments that promote the formation of VM channels [11, 12]

#### Notch

**Fig. 3:**
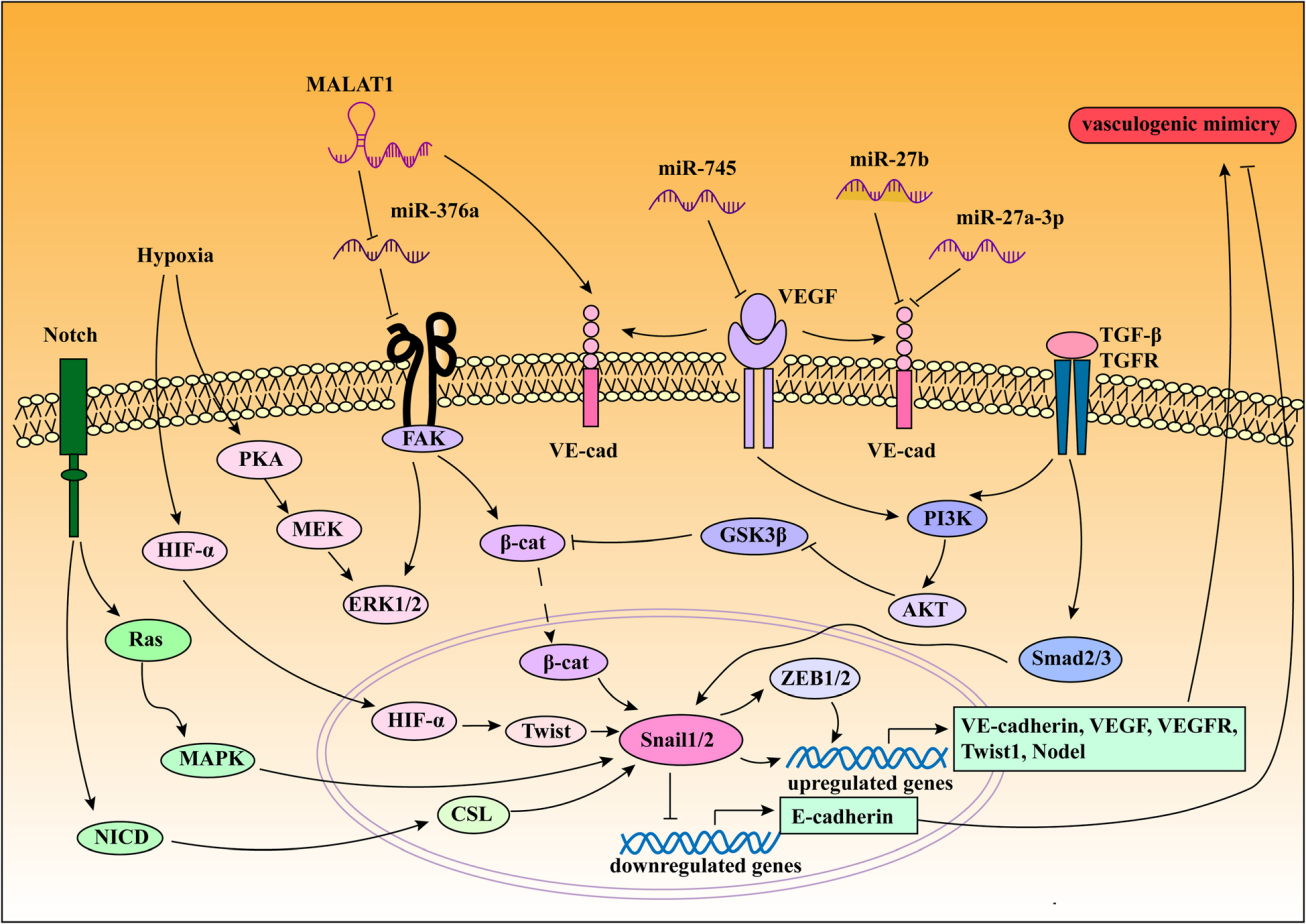
Complex mechanisms and signaling pathways underlying VM formation. (1) Hypoxia is one of the major important factors in VM formation and contributes to several crucial pathways by regulating expression of some signaling molecules, the expression of HIF-α is positively correlated with hypoxia target genes such as Twist, VEGF, and VEGFR. (2) lncRNA MALAT1 regulates VM formation via VE-cadherin, FAK/ERK signaling pathway, miR-27b and miR-27a-3p target VE-cadherin to regulate VM formation. (3) PI3K, AKT, and PKA are involved in the process of VM formation by PKA/MEK/ERK and TGF-β/PI3K/AKT axis. (4) Notch pathway mediates VM formation through the activation of Ras.

The Notch family, which is a transmembrane receptor consisting of four different isoforms (Notch1-4) and five possible membrane-bound ligands, Delta-like 1/3/4 and Jagged 1/2, is essential during the process of embryonic pluripotency and embryonic development. The Notch intracellular domain (NICD) is released into the cytoplasm and localized in the nucleus after the Notch receptor is sequentially degraded. The Notch signaling pathway was demonstrated to play a crucial role in the development of vascular networks, so recent studies revealed that Notch is associated with VM in malignant cancer. In melanoma, Vartanian et al. used γ-secretase inhibitors, DAPT, dibenzazepine or Jagged-1 neutralizing antibody to block Notch signaling, resulting in VM channel reduction [80]. An interesting study showed that the downregulation of Notch 4 disrupted VM network formation and inhibited the invasion and migration of tumor cells by inhibiting the activation of MMP-2 and MMP-9 in HCC [81]. Moreover, Notch 1 has been demonstrated to play a promotor role in HCC progression by activating the EMT pathway and forming VM channels [82]. In addition, extracts from Chinese herbs, such as Celastrus orbiculatus extract (COE) and Luteolin have been suggested to inhibit VM and tumor growth by downregulating Notch 1 signaling [69, 83] (Fig. 3).

### Non-coding RNAs, as emerging regulators in tumor VM formation

Non-coding RNAs (ncRNAs) are the functional RNA molecules that are not translated into proteins. ncRNAs can be grouped according to their length: those longer than 200 nucleotide called long non-coding RNAs (lncRNAs), those less than 200 nucleotide called small non-coding RNAs (small ncRNAs), and those less than 50 nucleotide can also be called tiny ncRNAs, such as siRNAs, miRNAs, and piRNAs. Recently, emerging evidence has demonstrated that lncRNAs [16, 70, 71, 73] and miRNAs [74, 75, 84, 85] play an important role in the process of VM formation. Next, we will discuss how lncRNAs and miRNAs regulate in VM formation.

Glioma has the strong ability of VM formation. In 2016, Xue et al. reported that microRNA-Let-7f (miR-Let-7f) functions as a tumor suppressor by inhibiting VM [86]. Further study revealed that miR-Let-7f suppresses the VM forming capacity of glioma by disturbing POSTN-dependent migration. It is demonstrated that POSTN is a direct target of miR-Let-7f in the process of VM formation. microRNA-584-3p (miR-584-3p) was also reported to disturb hypoxia-induced stress fiber formation to suppress VM formation. Their results demonstrated that ROCK1 can promote the formation of stress fibers in an anoxic environment is a potential functional target of miR-584-3p. As a result, miR-584-3p antagonized hypoxia-induced ROCK1-dependent stress fiber formation to inhibit the VM of tumor cells [87]. In primary gliomas, the expression of miR-141 is downregulated and negatively correlated with VM density. In addition, EphA2, a potential direct target of miR-141, has a negative correlation with the expression levels of miR-141. VM formation was inhibited by miR-141 by controlling EphA2 expression [88]. Guo et al. reported that LINC00339 was upregulated in human glioma and positively correlated with VM formation. Bioinformatics and luciferase reporter assays showed that LINC00339 regulates VM by binding to miR-539-5p. Further study revealed that Twist1 binds to the promoters of MMP-2 and MMP-14 genes to regulate the tumor-suppressive effects of miR-539-5p. In conclusion, LINC00339 regulates VM formation by regulating the miR-539-5p/Twist1/MMP-2/MMP-14 pathway in glioma [89]. Long coding RNA HOXA-AS2 was upregulated and was correlated with VM in glioma samples. The expression of VE-cadherin, MMP-2, and MMP-9 was inhibited after HOXA-AS2 knockdown, according to the study, miR-373 was downregulated in glioma samples, but the expression of miR-373 was upregulated after HOXA-AS2 knockdown. Dual-luciferase reporter assays and RNA immunoprecipitation (RIP) assays showed that HOXA-AS2 can bind to miR-373 in a sequence-specific manner. Further study showed that miR-373 regulates VE-cadherin, MMP-2, and MMP-9 through targeting EGFR. In addition, EGFR activated PI3K/serine/threonine kinase pathways to increase the expression of VE-cadherin, MMP-2, and MMP-9. In conclusion, HOXA-AS2 promoted malignant glioma progression and VM formation through the miR-373/EGFR axis [90].

In HCC, downregulated miR-27a-3p is associated with metastasis and VM, and further experiments validate that miR-27a-3p inhibits metastasis and VM formation by targeting the 3′-UTR of VE-cadherin, downregulating its expression and suppressing EMT signaling [91, 92]. In 2016, Yang et al. reported that miR-101 targets TGF-ΒR1 and Smad2 to attenuate TGF-β signal transduction in tumor cells and blocks SDF1 signaling by suppressing the expression of SDF1 and VE-cadherin. This study revealed that miR-101 regulates VM formation via the TGF-β/SDF1-VE-cadherin/MMP-2 network [93]. It has been reported that CSCs play a crucial role in VM formation. Zhao et al. found that lncRNA n339260 is associated with CSC in HCC, and that overexpression of n339260 induced a significant increase in VM formation and expression of VE-cadherin. They used Affymetrix miRNA Chip 4.0 to predict the downstream miRNAs regulated by n339260, and miR-31-3p, miR-30e-5p, miR-519c-5p, miR-520c-5p, miR-29b-1-5p, and miR-92a-1-5p were identified [68].

In triple-negative breast cancer (TNBC), lncRNA TP73-AS1 was upregulated and associated with VM density in cancer tissues. Further experiments revealed that TP73-AS1 modulates TNBC cell VM formation by binding to miR-490-3p. Twist1 as a direct and specific target of miR-490-3p can accelerate VM formation [94]. In 2018, Salinas-Vera et al. showed that miR-204 regulates VM formation by targeting the PI3K/AKT/FAK signaling pathway [95]. It has been reported that miRNAs such as miR-193b, niR-125a, and let-7e suppress VM formation in breast cancer. Park et al. found an inverse correlation between miR-125a/let-7e and IL-6 after cisplatin treatment. Furthermore, IL-6-induced adhesion of monocytes to ECs and VM formation were suppressed by endothelial miR-125a/let-7e [96]. miR-193b regulates breast cancer cells (MDA-MB-231 cells) migration and VM formation via targeting dimethylarginine dimethylaminohydrolase 1 (DDAH1) [97] (Table 1).

**Table 1:** MiRNAs in VM

MiRNA	Cancer type	Target	Function characteristic	Effect of VM formation	Reference
miRNA-27b	Ovarian cancer	VE-cadherin	inhibits ovarian cancer cell migration and VM via binding to the 3′-untranslated region(3′UTR) of VE-cadherin mRNA	Suppress	[98]
miRNA-584-3P	Glioma	ROCK1	Disturbs hypoxia-induced ROCK1-dependent stress fiber formation	Suppress	[87]
MiRNA-let-7f	Glioma	POSTN	Disturbs the POSTN-dependent migration	Suppress	[86]
miR141	Glioma	EphA2	Inhibits EphA2 expression	Suppress	[88]
miR-27a-3p	Hepatocellular carcinoma	VE-cadherin	Targets the 3′-UTR of VE-cadherin, and suppresses EMT signaling	Suppress	[92]
miR-193b	Breast cancer	DDAH1	Regulates MDA-MB-231 cells migration and VM formation via targeting DDAH1	Suppress	[97]
miR-125a/let-7e	Breast cancer	IL-6, IL-6R, STAT3	Suppresses IL-6-induced adhesion of monocytes to ECs and VM formation	Suppress	[96]
miR-200a	Ovarian cancer	EphA2	Inhibits EphA2 expression	Suppress	[99]
miR-490-3p	Clear cell renal cell carcinoma	Vimentin	-	Suppress	[28]
miR-745	Ovarian cancer	VEGFR/AKT1/SRC-α	Decreases the levels of VEGFA, AKT1, and SRC-α transducers and exerts a negative regulation of VEGFA by specific binding to its 3'UTR	Suppress	[74]
miR-9	Glioma	Stathmin (STMN1)	targets the 3′-UTR of STMN1	Suppress	[75]
miR-186	Prostate cancer	Twist1	-	Suppress	[84]
miR-101	Hepatocellular carcinoma	TGF-β1, Smad2	Targets TGF-ΒR1 and Smad2 to attenuate TGF-β signaling transduction in tumor cells and blocks SDF1 signaling	Suppress	[93]
miR-124	Cervical cancer	Amotl1	Represses VM, migration, and invasion in HeLa and C33A cells by regulating AmotL1’s 3'untranslated region (3'UTR)	Suppress	[85]

MALAT1, as a well-characterized lncRNA molecule, plays an important role in cancer cell invasion and metastasis and VM formation. In gastric cancer, the expression of MALAT1 was associated with VM density and endothelial vessels. Functional experiments revealed that MALAT1 can regulate expression of classical VM markers, such as VE-cadherin, β-catenin, MMP-2/9, MT1-MMP, p-ERK, p-FAK and p-paxillin, and MALAT1, whose levels were also associated with OS in stage gastric cancer patients. This finding suggests that MALAT1 regulates VM formation via the VE-cadherin/β-catenin complex, and the ERK/MMP and FAK/paxillin signaling pathways [16]. In lung adenocarcinoma, LINC00312 was found to regulate migration, invasion, and VM processes of lung cancer cells. LINC00312 directly binds to the transcription factor Y-box binding protein 1 (YBX1) to regulate VM formation [26]. In ovarian cancer, miR-200a and miR-27b have been reported to be significantly downregulated, and their expression levels were inversely correlated with VM formation. miR-200a negatively regulated EphA2 expression to inhibit VM(99). miR-27b inhibited ovarian cancer cell migration and VM by binding to the 3′-untranslated region(3′UTR) of VE-cadherin mRNA, which led to suppression of the VM formation [98] (Table 2).

**Table 2:** LncRNAs in tumor VM formation

LncRNA	Cancer type	Cancer cell lines	miRNA	Signaling pathway	Effect of VM formation	Reference
MALAT1	Gastric cancer	BGC823 SGC7901	miR-376a	ERK/MMP FAK/paxillin pathway	Promote	[16, 71]
MALAT1	Lung cancer	H1299 H292	miR-145-5p	lncRNA-MALAT1/miR-145-5p/NEDD9 pathway	Promote	[70]
LINC00339	Glioma	U87 U251	miR-539-5P	miR-539-5P/Twist1/MMPs pathway	Promote	[89]
LncRNA n339260	Hepatocellular carcinoma	HepG2	-	TGF-β pathway	Promote	[68]
LncRNA HOXA-AS2	Glioma	U87 U251	miR-373	miR-373/EGFR pathway	Promote	[90]
LncRNA TP73-AS1	Triple negative breast cancer	MDA-MB-231	miR-490-3P	miR-490-3p/Twist1 pathway	Promote	[94]
LINC00312	Lung adenocarcino-ma	H1299 PC-9	-	YBX1/AKT/TGF-β pathway	Promote	[26]
SNHG20	Glioma	U87 U251	-	ZRANB2/SNHG20/FOXK1 pathway	Promote	[73]

In conclusion, increasing evidence suggests that lncRNAs and miRNAs play a critical role in malignant tumor VM formation, but whether other non-coding RNAs besides lncRNAs and miRNAs regulate VM formation has not yet been investigated in malignant tumors. The detailed mechanisms of VM formation need to further in-depth studies.

#### Clinical significance of VM in cancer

Ample evidence indicates that VM is significantly associated with poor overall survival in patients, suggesting that VM potentially indicates a poor prognosis for patients with malignant tumors, such as osteosarcoma [100], gliomas [45], breast cancer [63, 95], gastric cancer [16], HCC [11, 68], and lung cancer [19, 101]. It is known that VM is associated with tumor growth, progression, metastasis, invasion, and treatment failure. According to many studies, patients with VM-positive tumors have a worse prognosis and a lower 5-year survival rate than patients with VM-negative tumors [11, 16, 70]. VM has been demonstrated as a potential independent indicator of poor prognosis, and the identification of VM is very important to clinical practice.

##### Histologic and imaging characteristics of VM

VM has been demonstrated as a potential indicator of the poor prognosis of patients with VM-positive tumors in many studies [3, 16, 20, 26, 100, 102, 103]. Immunohistochemistry (IHC) staining is currently the gold standard for VM diagnosis in malignant tumors. VM exhibits PAS-positive but CD31-negative vascular-like channels. However, IHC staining has some limitations. Color Doppler imaging was reported to distinguish the blood flow of endothelial-lined and tumor cell-lined vasculatures. Wolfram Ruf et al. used color Doppler ultrasound and microbubble imaging to study the blood flow of aggressive human cutaneous melanoma xenografts, and color Doppler revealed abundant blood flow in tumor cell-lined channels. Additionly, tracking of microbubbles showed the connection between endothelial-lined and tumor cell-lined vasculatures [104]. Magnetic resonance imaging (MRI) is a critical tool in the diagnosis of cerebral cavernous malformations (CCMs), but it is insufficient to distinguish between CCMs and VM because of the spatial resolution limitations of MRI. In the study, the authors found VM revealed a rim with a weak and dark intensity when compared to CCMs by using fluid-attenuated inversion recovery (FLAIR) images [105]. In the early stage of tumors, it is very difficult to detect and diagnose cancer because patients have no symptoms. Thus, the development of novel molecular imaging technologies is essential for the early stage tumor detection and diagnosis. Molecular imaging could be used to understand the biological processes of VM in living organisms. Interestingly, specific contrasting agents that can enter VM tubes and be detected by novel molecular imaging technologies can provide non-invasive imaging for a better way to detect VM in patients, which can guide treatment [20, 106, 107]. However, there are many challenges, such as absence of specific contrasting agent that could distinguish between blood vessels formed via VM or tumor angiogenesis, and the ability of different malignant tumors to form VM tubular structures is different. Moreover, there are still a lot of debates on the identification of VM structures and a lack of common standards. At present, most of the research on applying molecular imaging technology to VM is limited to animals. Thus, further exploration and development are needed to make remarkable progress, and for molecular imaging technology of VM to become a tool for clinical application.

##### Potential therapeutic targeting of VM

Anti-angiogenic therapy has become an effective component of the treatment of malignant tumors. Currently available anti-angiogenic drugs mainly inhibit endothelial cell proliferation or induce endothelial cell apoptosis, decrease vascular density, and cause hypoxia in the tissue. Anti-angiogenic molecules such as VEGFR1, thrombospondins, and semaphorins could antagonize pro-angiogenic molecules such as VEGF and Ang2. The current anti-angiogenic drugs such as bevacizumab, sunitinib, sorafenib, aflibercept, axitinib, pazopanib, and regorafenib are applied to the treatment of tumor patients [30, 108–110]. However, anti-angiogenic drugs cannot effectively stop the neovascularization process because hypoxia promotes the formation of VM, and the formation of VM could reduce the therapeutic effect. Many studies have attempted to inhibit tumor VM specifically. In TNBC, brucine, a traditional medicinal herb, was reported to inhibit VM formation in a dose-dependent manner, and downregulate the expression of EphA2, MMP-2, and MMP-9, which are key mediators of tumor invasion, metastasis, and VM formation [111]. Furthermore, flavone isoxanthohumol and tratuzumab have been demonstrated as inhibitors in the process of VM formation. Serwe et al. found that flavone isoxanthohumol decreases the formation of VM by blocking the IFN-γ, IL-4, and IL-6 dependent Jak/Stat signaling [112]. Trastuzumab, a drug that targets the receptor tyrosine kinase HER2, might inhibit VM in the HER2-positive BCC microenvironment [113]. Similar approaches have been reported for VM in melanoma. An interesting study by Kumar et al. has showed that tivantinib alters proteins such as vinculin and RhoC to affect the cell cytoskeleton and morphology, and thereby decreases VM formed by melanoma cells in a 3D matrix [114]. Various traditional Chinese medicine have been reported to play critical roles in the treatment of malignant cancers. R8-modified epirubicin-dihydroartemisinin liposomes have been reported to suppress VM through regulating the levels of VE-cad, TGF-β, MMP-2, and HIF-1α in non-small-cell lung cancer. Epirubicin is an anti-tumor drug most commonly applied in the treatment of various cancer types, and dihydroartemisinin is an anti-malarial drug but also has anti-tumor effects [115]. Additionally, ginsenoside Rg3 (Rg3) is a tetracylic triterpenoid saponin that inhibits tumor cell proliferation, adhesion, and tumor angiogenesis. Guo et al. revealed that the decrease of VM and the expression of VE-cadherin, EphA2, MMP-2, and MMP-9 in both Rg3-treated tumor xenografts and vitro cells [116]. Moreover, dioscin is an extract from traditional medicinal plants and showed as an inhibitor in the process of VM formation in daunorubicin and dioscin codelivery liposomes [117]. Melittin has been confirmed as an inhibitor in the process of tumor angiogenesis and metastasis. In the study, melittin inhibits liver cancer through suppressing hypoxia-induced VM formation [118]. Both thalidomide and rapamycin target VEGF to inhibit tumor VM formation [119, 120]. VEGF, a vascular permeability factor, is thought to be a critical regulator of tumor angiogenesis. Some anti-VEGF drugs such as bevacizumab, sunitinib, regorafenib, sorafenib, aflibercept have been widely applied in the treatment of various types of malignant cancer. There are several anti-VM therapeutic agents [121–123] such as dequalinium (DQA) modified paclitaxel plus ligustrazine micelles, thalidomide, trastuzumab, tapamycin, resveratrol, and resveratrol that inhibit VM by targeting VEGF. A combination of VM-targeting and endothelium-targeting anti-angiogenic drugs can block the blood supply of tumors and inhibit the growth of tumors more completely and efficiently than each agent alone [124] (Table 3).

**Table 3:** Therapeutic agents targeting VM

Pharmacological agents	Cancer type	Molecule target	Drug action	Referrence
Brucine	Triple-negative breast cancer	EphA2/MMP-2/MMP-9	Suppresses VM by disrupting F-actin cytoskeleton and microtubule structure	[111]
R8 modified epirubicin-dihydroartemisinin liposomes	Non-small-cell lung cancer	VE-cadherin/TGF-β/MMP-2/HIF-1	Suppresses VM channels and tumor metastasis by downregulating the levels of VE-cad, TGF-β, MMP-2 and HIF-α	[115]
Ginsenoside Rg3	Pancreatic cancer	VE-cadherin/EphA2/MMP-2/MMP-9	Downregulates the levels of VE-cad, EphA2, MMP-2 and MMP-9 to inhibit the formation of VM	[116]
Dequalinium (DQA) modified paclitaxel plus ligustrazine micelles	Non-small-cell lung cancer	VEGF/MMP-2/ TGF-β/E-cadherin	Destroies VM channels and down regulate the expression of VEGF, MMP-2, TGF-β and E-cadherin	[122]
Favone isoxanthohumol	Breast cancer	IFN-γ/IL-4/IL-6 Jak/Stat signaling	Blocks IFN-γ/IL-4/IL-6 Jak/Stat signaling and TGF-β signaling to inhibits VM formation	[112]
Dunorubicin and dioscin codelivery liposomes	Non-small-cell lung cancer	MMP-2/VE-cadherin TGF-β/HIF-1α	Inhibits VM formation by down regulating the levels of MMP-2, VE-cadherin, TGF-β and HIF-1α	[117]
Melittin	Liver cancer	HIF-1α/Akt	Inhibits hypoxia-induced VM formation and EMT by suppressing HIF-α/Akt pathway	[118]
Tivantinib (TivB)	Melanoma	Vnculin/RhoC	Disrupts VM channels by targeting vinculin and RhoC	[114]
PARP inhibition	Melanoma	VE-cadherin	Inhibits reduced pro-metastatic markers	[110]
Thalidomide	Melanoma	VEGF/NF-ΚB/MMP-2/MMP-9	Regulates vasculogenic factors to inhibit VM channel and mosaic vessels formation	[119]
Trastuzumab	HER2-positive breast cancer	VEGF	Suppresses VM in HER2-positive BCCs	[113]
Rapamycin	Ovarian cancer	VEGF/mTOR	As HIF-α inhibitor to prevent VM	[120]
Resveratrol	Melanoma	VEGF-R1/VEGF-R2	Suppresses VM formation	[121]
Niclosamide	Oral cancer	miR-124/STAT3	Inhibits VM formation through downregulation of the expression of VM-related genes VEGFA, MMP2, ROCK1, and Cdc42	[123]
Celastrus orbiculatus extrac (COE)	Hepathocellular carcinima	Notch1 signaling	Inhibits VM formation by downregulating Notch 1 signaling	[83]
Luteolin	Gastric cancer	Notch 1/VEGF	Inhibits VM formation through suppressing VEGF secretion dependent on Notch1 expression	[69]

Recently, further insight into the molecular signaling that triggers and promotes VM formation could improve anti-angiogenic therapeutics. Several genes such as MMP-2, MT1-MMP, FAK, TGF-β, and VE-cadherin could be potential targets for therapy. Furthermore, CSCs also play a significant role in the design of novel anti-tumor therapies [11–13]. The latest findings reveal that lncRNAs such as LINC00339, LINC00312, and MALAT1 could also be promising targets in anti-VM therapy [16, 26, 77]. Therefore, the clinical application of therapeutic strategies by targeting tumor VM needs further investigation in the future.

## Conclusion and perspectives

In this review, we summarized recent research advances in VM formation in tumors and versatile mechanisms that regulate tumor VM formation. Notably, increasing evidence indicates that VM plays a crucial role in tumor invasion, metastasis, and poor prognosis in patients with malignant tumors. VM is a potential novel target of anti-tumor therapy. However, the mechanisms by which VM is promoted have not been fully elucidated. Emerging evidence suggests that non-coding RNAs (lncRNAs and miRNAs) play a critical role in malignant tumor VM formation, but the mechanisms of lncRNAs and miRNAs in the process of VM formation remain unclear. Recently, some studies have shown that molecular imaging technologies provide an excellent way to detect whether a tumor is VM positive, but they are limited to animals. More exploration and development are needed to make molecular imaging technologies be a diagnostic tool for clinical application. It is well known that anti-angiogenesis therapy remains unsatisfactory because of the formation of VM. Some pharmacological agents targeting VM can potentially inhibit the tumor progression effectively. Therefore, the clinical application of new agents targeting tumor VM with specific molecular compounds needs further exploration. The combinatorial application of VM-targeted drugs, endothelium-targeted drugs, and other therapies might hold a great promise in cancer therapy.

## Data Availability

The materials supporting the conclusion of this review have been included within the article.

## Abbreviations

3′UTR: 3′-untranslated region
AML: Acute myeloid leukemia
BCCs: Breast cancer cells
CAFs: Cancer-associated fibroblasts
cAMP: Cyclic adenosine monophosphate
CCMs: Cerebral cavernous malformations
CDK5: Cyclin-dependent kinase 5
CDX: CTC patient-derived explants
CEACAM6: Carcinoembryonic antigen-related cell adhesion molecule 6
CM-TAM: Conditioned medium of tumor-associated fibroblast
CSCs: Cancer stem cells
CTCs: Circulating tumor cells
DDAH1: Dimethylarginine dimethylaminohydrolase 1
DQA: Dequalinium
EC-like TCs: Endothelial cell-like tumor cells
ECs: Endothelial cells
EMT: Epithelial-mesenchymal transition
EMT-TFs: Epithelial-mesenchymal transition-transcription factors
EPC: Endothelial progenitor cell
EphA2: Erythropoietin-producing hepatocellular receptor A2
ER: Endoplasmic reticulum
ER: Estrogen receptor
FAK: Focal adhesion kinase
FAP: Fibroblast-activated proteins
FLAIR: Fluid-attenuated inversion recovery
GBM: Glioblastoma multiforme
GSCs: Glioblastoma stem like cells
GSK3β: Glycogen synthase kinase-3β
HCC: Hepatocellular carcinoma
HER: Hypoxia responsive elements
HER2: Human epidermal growth factor receptor 2
HGSOC: High-grade serous ovarian cancer
HIF-1α: Hypoxia inducible factor-1a
HNSCC: Head and neck squamous cell carcinoma
IA: Intussusceptive angiogenesis
IHC: Immunohistochemistry
LAMC2: Laminin subunit 5 gamma-2
lncRNAs: Long non-coding RNAs
miRNAs: MicroRNAs
MMP-2: Matrix metalloproteinase-2
MMPs: Matrix metalloproteinases
MRI: Magnetic resonance imaging
MT1-MMP: Membrane type 1 matrix metalloproteinase
ncRNAs: Non-coding RNAs
pEGFR: Phosphorylated EGFR
PI3K: Phosphatidyl inositol 3-kinase
PR: Progesterone receptor
pVEC: PhosphoVEC
RBCs: Red blood cells
Rg3: Ginsenoside Rg3
RIP: RNA immunoprecipitation
ROS: Reactive oxygen species
RTKs: Tyrosine kinase receptor
SAM: Scanning electron microscopy
SCID: Severe combined immune deficiency
TAF: Tumor-associated fibroblast
TAM: Tumor-associated macrophage
TCs: Tumor cells
TEM: Transmission electron microscopy
TGF-β: Transforming growth factor-beta
TNBC: Triple-negative breast cancer
VE-cadherin: Vascular endothelial-cadherin
VEGF: Vascular endothelial growth factor
VEGFR1: Vascular endothelial growth factor receptor
VM: Vasculogenic mimicry
YBX1: Y-box binding protein 1
ZEB1: Zinc finger E-box binding homeobox 1
ZEB2: Zinc finger E-box binding homeobox 2
